# Supplementary material for: Anionic polymerization of nonaromatic maleimide to achieve full-color nonconventional luminescence
Source: Nat Commun. 2022 Jun 28;13:3717. doi: 10.1038/s41467-022-31547-2 (PMC9240025; doi:10.1038/s41467-022-31547-2)
Supplement: Supplementary file 1 — Supplementary Information [file 41467_2022_31547_MOESM1_ESM.pdf]

Supplementary Information

Anionic polymerization of nonaromatic maleimide to achieve full-color nonconventional luminescence

Xin Ji[1,2], Weiguo Tian[1*], Kunfeng Jin[1], Huailing Diao[1,2], Xin Huang[3], Guangjie Song[1*], Jun Zhang[1,2*]

[1] Beijing National Laboratory for Molecular Sciences, CAS Key Laboratory of Engineering Plastics, Institute of Chemistry Chinese Academy of Sciences (CAS), Beijing 100190, China; [2] University of Chinese Academy of Sciences, Beijing 100049, China; [3] School of Chemical Engineering and Technology, Tianjin University, Tianjin 300072, China. E-mail: wgtian@iccas.ac.cn; gsong@iccas.ac.cn; jzhang@iccas.ac.cn.

Content of Supplementary Information

Supplementary Figures P. 3

**Figure 1** Excitation and emission spectra of Fr-PM-AIBN	P. 3
**Figure 2** Large-scale production of A-PMs powders in laboratory	P. 3
**Figure 3** Chemical structure characterization of different PMs	P. 4
**Figure 4** GPC analysis of different PMs	P. 4
**Figure 5** Thermal analysis of different PMs	P. 5
**Figure 6** Images of different PM powders under visible and UV light	P. 5
**Figure 7** Emission properties of different PMs solutions and powders	P. 5
**Figure 8** GPC analysis of A-PM-TEA at different reaction time	P. 6
**Figure 9** Spectroscopic study of the polymerization kinetics for A-PM-TEA	P. 6
**Figure 10** Emission of A-PM-TEA synthesized at different temperature	P. 6
**Figure 11** Concentration-dependent emission of different A-PMs	P. 7
**Figure 12** Spectroscopic study of A-PM-TEA in DMF	P. 8
**Figure 13** Solvent-dependent emission of A-PM-TEA	P. 8
**Figure 14** Temperature-dependent emission of A-PM-TEA	P. 9
**Figure 15** Temperature-dependent fluorescent images of A-PM-TEA powder	P. 9
**Figure 16** Multi-peak fitting analysis of the emission spectra during compression	P. 10
**Figure 17** Multi-peak fitting analysis of the emission spectra during decompression	P. 11
**Figure 18** Detailed multi-peak fitting analysis for compression process	P. 12
**Figure 19** Detailed multi-peak fitting analysis for decompression process	P. 12
**Figure 20** Optimized molecular conformation and dipole moment of PMs	P. 13

**Figure 21** Excited-state HOMO/LUMO of PMs	P. 13
**Figure 22** Emission change of A-PM-TEA after hydrolysis	P. 14
**Figure 23** Excitation-emission contour plots of A-PMs	P. 15
**Figure 24** Fluorescent response of A-PM-TEA to Fe^{3+}	P. 16
**Figure 25** Mechanism for the emission of A-PM-TEA quenched by Fe^{3+}	P. 16
**Figure 26** Emission of A-PM-TEA with TEA	P. 17
**Figure 27** Emission of Fr-PM-AIBN with TEA	P. 17
**Figure 28** Emission changes of Fr-PM-AIBN/DMF solutions over the time after adding TEA	P. 17
**Figure 29** ^{1}H NMR and FTIR analysis of Fr-PM-AIBN before and after mixing with TEA	P. 18
**Figure 30** Proposed synthetic routes of A-PM-HA and A-PM-PMP	P. 18

Supplementary Tables **P. 19**

**Table 1** GPC results of different PMs	P. 19
**Table 2** PLQY % of different A-PM powders	P. 19
**Table 3** GPC analysis of the polymerization kinetics for A-PM-TEA	P. 19
**Table 4** PLQY of A-PM-TEA/TEA mixtures	P. 19
**Table 5** Synthetic information of the samples presented in the main text	P. 20

Supplementary Methods **P. 21**

**Cell viability assay for skin-contact compatibility**	P. 21
**Fluorescent pattern printing**	P. 21
**Metal ions detection**	P. 21
**Latent fingerprint detection**	P. 22

Supplementary Discussion **P. 23**

**Through space charge transfer (TSCT), intermolecular charge transfer (ICT) and internal conversion (IC)**	P. 23
**Mechanism for the emission of A-PM-TEA quenched by Fe^{3+}**	P. 24
**Emission of A-PM-TEA and Fr-PM-AIBN with the residual TEA**	P. 24

Supplementary References **P. 25**

Supplementary Figures

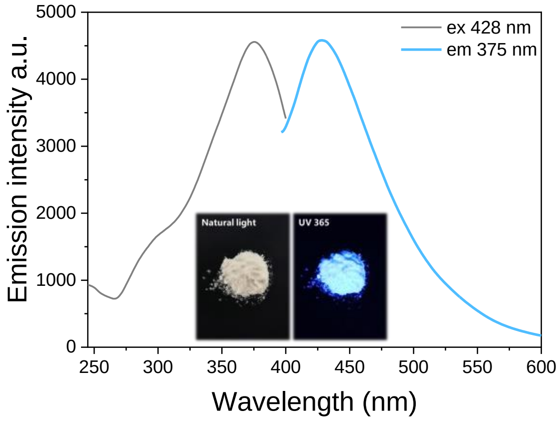

Figure 1 Excitation and emission spectra of Fr-PM-AIBN. Excitation spectrum, black line, EM WL = 428 nm; emission spectrum, blue line, EX WL = 375 nm.

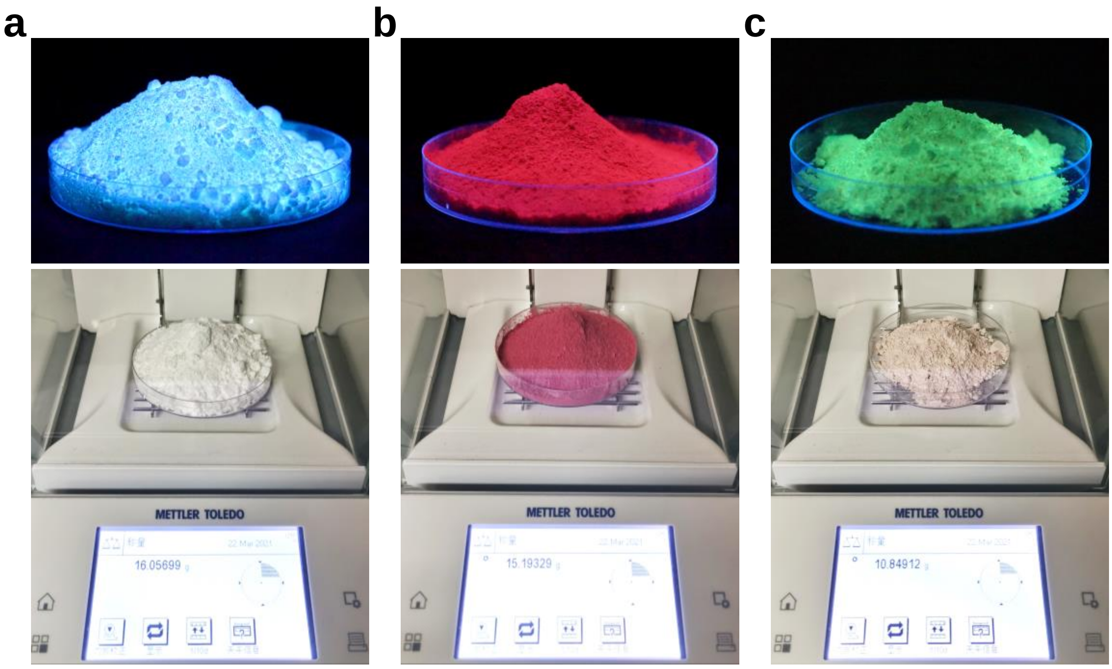

Figure 2 Large-scale production of A-PMs powders in laboratory. (a) A-PM-HA; (b) A-PM-TEA; (c) A-PM-PMP.

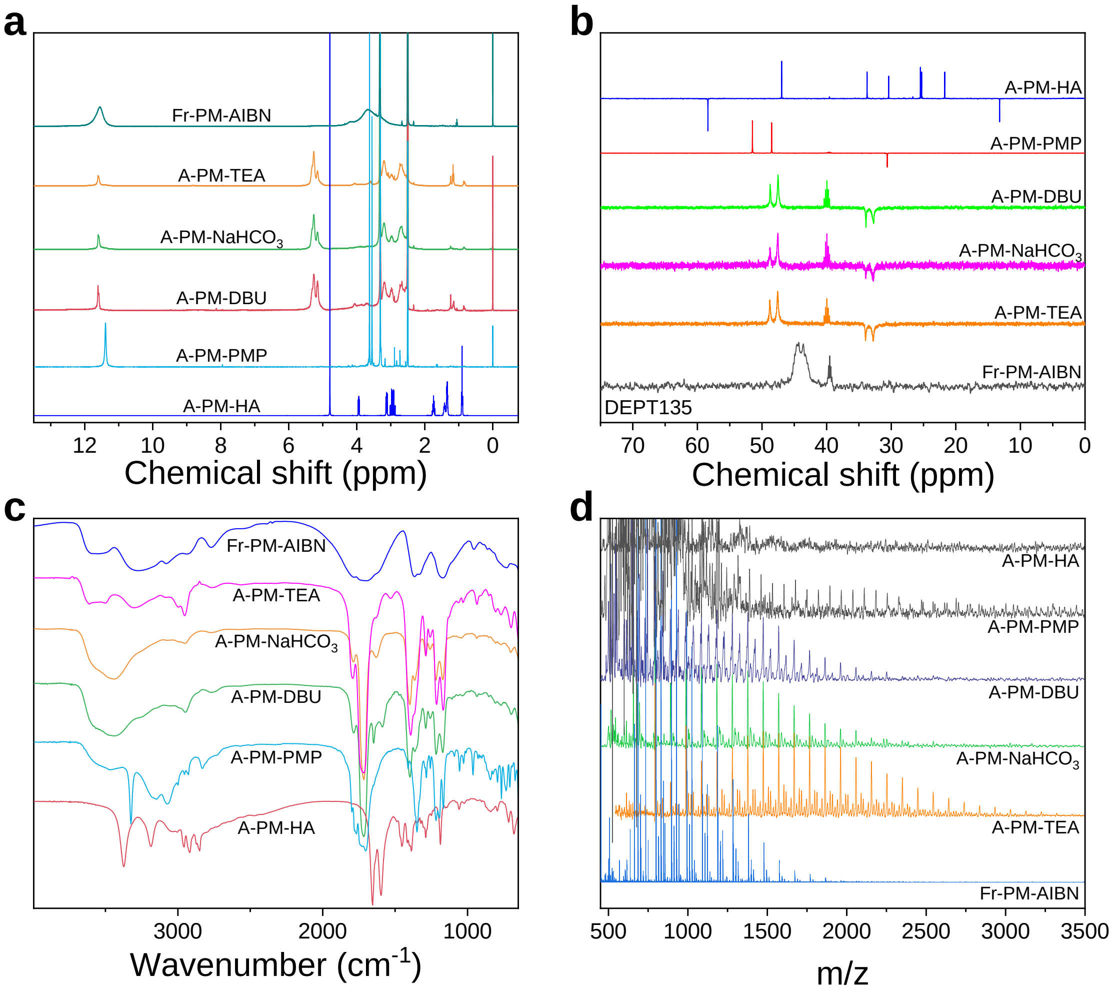

Figure 3 Chemical structure characterization of different PMs. a ^{1}H NMR, **b** DEPT-135 ^{13}C NMR, **c** FTIR, and **d** MS spectra

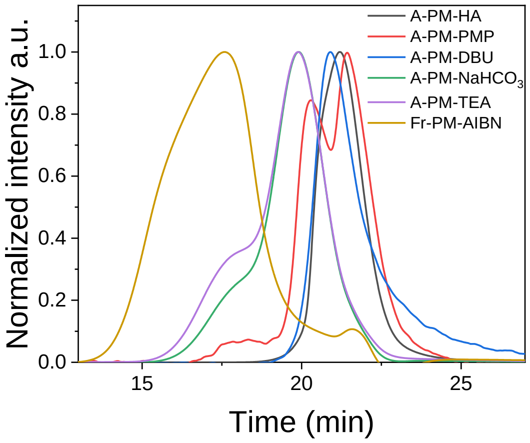

Figure 4 GPC analysis of different PMs. Number-average molecular weight (Mn), weight-average molecular weight (Mw), polydispersity (Mw/Mn) were provided in Table 1.

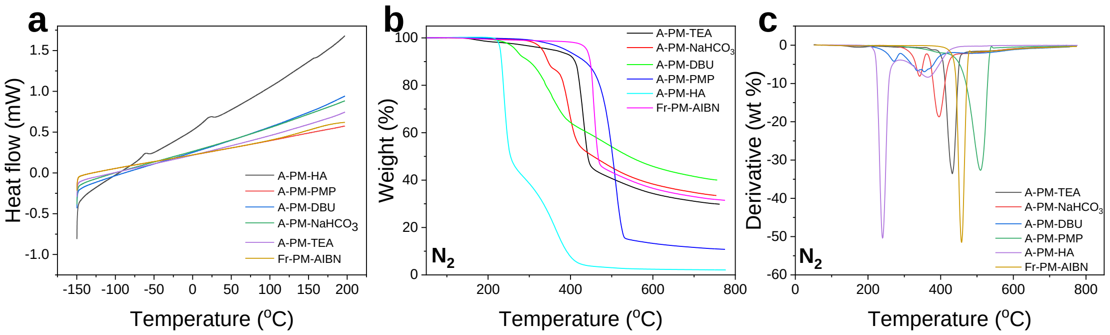

Figure 5 Thermal analysis of different PMs. a DSC, **b** TG, **c** DTG.

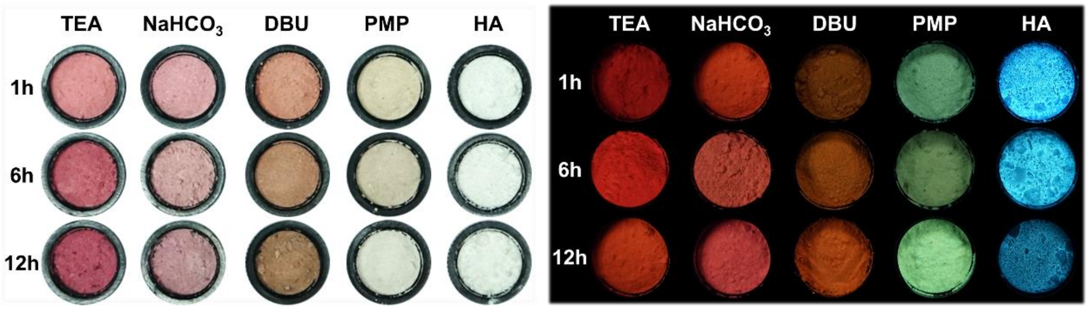

Figure 6 Images of different PM powders under visible and UV light. Left, nature light; right, UV 365 nm.

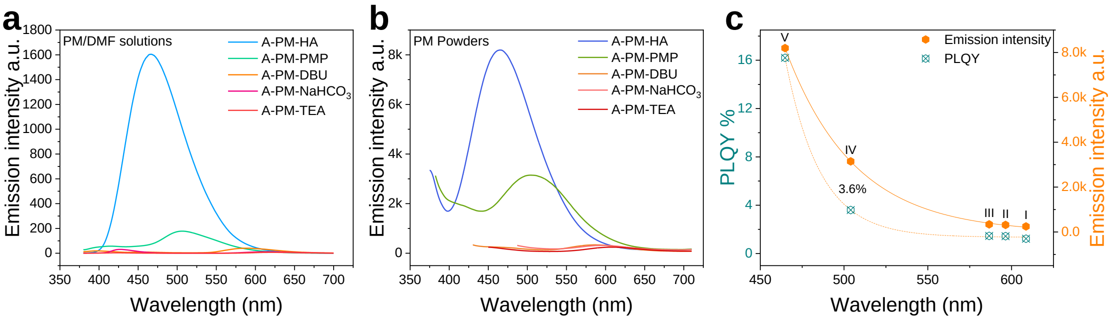

Figure 7 Emission properties of different PMs solutions and powders. a Emission spectra of PMs/DMF solutions. **b** Emission spectra of PMs powders (EX WL 365 nm). **c** Emission intensity and related PLQY of PM powders synthesized with different Lewies bases (I, TEA; II, $NaHCO_3$; III, DBU; IV, PMP; V, HA).

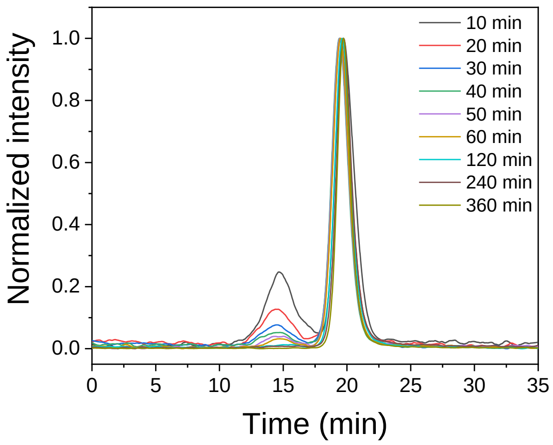

Figure 8 GPC analysis of A-PM-TEA at different reaction time. 80 °C, 10-360 min. Number-average molecular weight (Mn), weight-average molecular weight (Mw), polydispersity (Mw/Mn) were provided in Table 3.

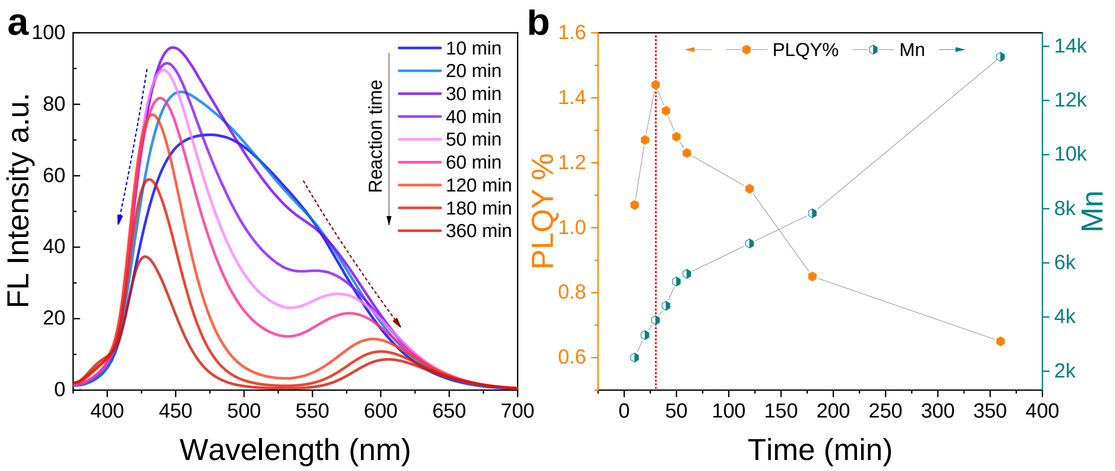

Figure 9 Spectroscopic study of the polymerization kinetics for A-PM-TEA. a *in-situ* emission spectra over different reaction time. **b** PLQY (%). Lewis base, TEA; 80 °C, 10-360 min.

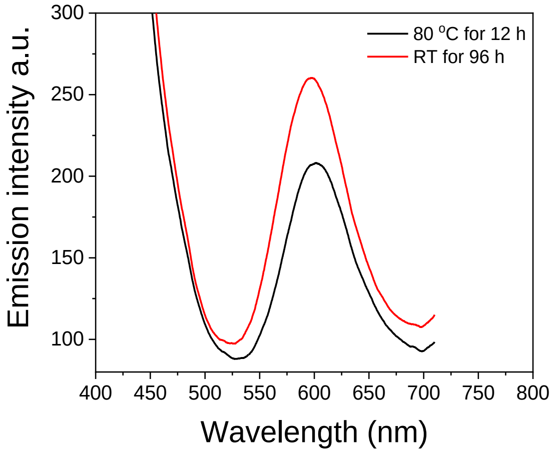

Figure 10 Emission of A-PM-TEA synthesized at different temperature. 80 °C and room temperature.

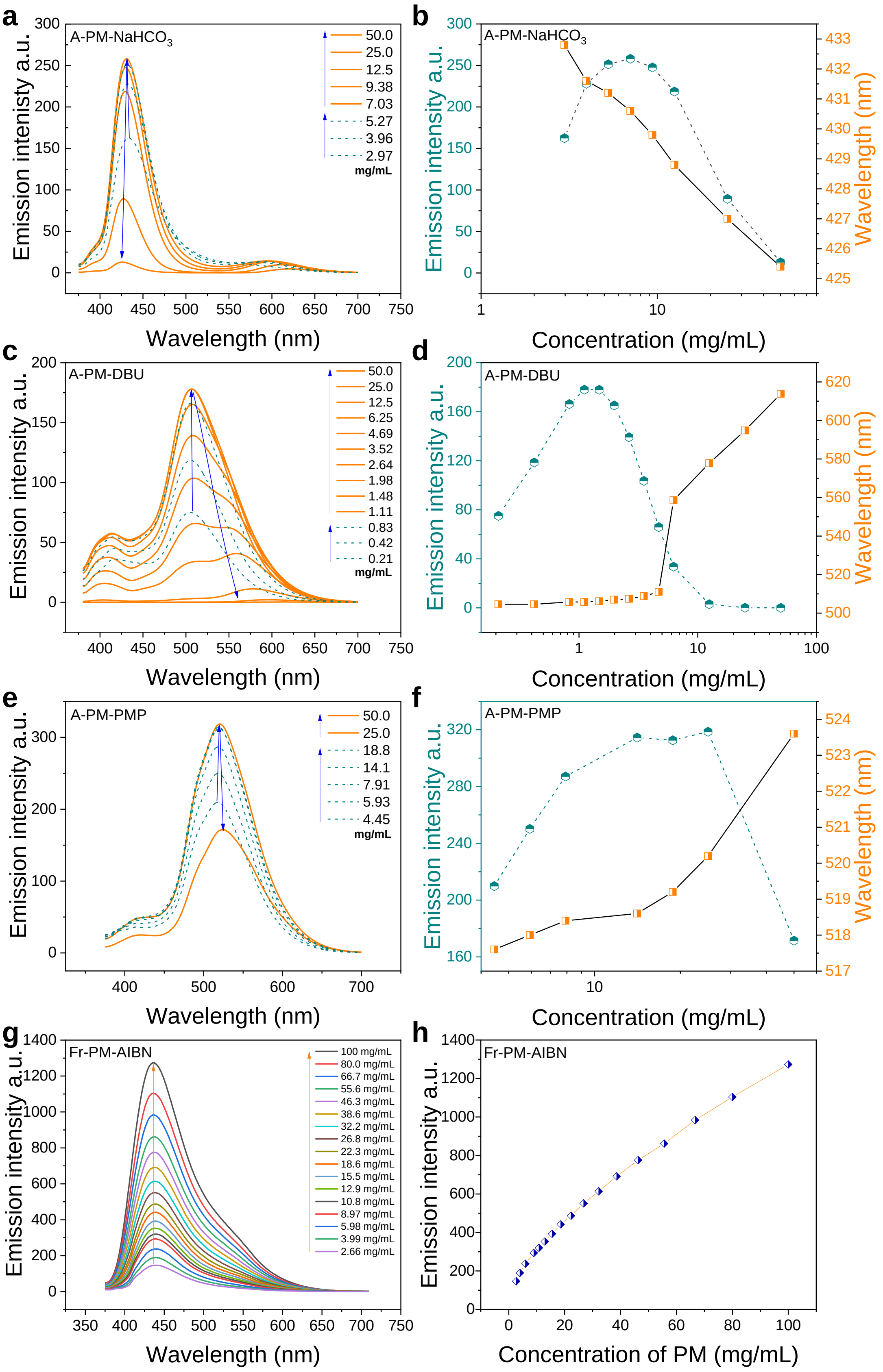

Figure 11 Concentration-dependent emission of different A-PMs. a b A-PM-$NaHCO_3$, **c d** A-PM-DBU, **e f** A-PM-PMP and **g h** Fr-PM-AIBN, including the emission spectra, intensity (at λ_{max}) and λ_{max} changes in DMF with different concentrations.

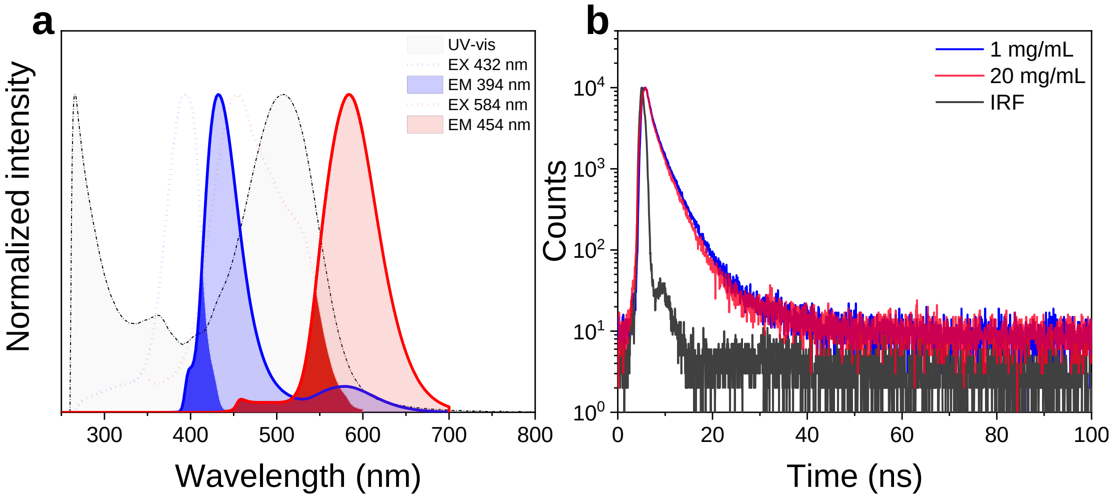

Figure 12 Spectroscopic study of A-PM-TEA in DMF. a UV-Vis, excitation and emission spectra. **b** fluorescence decay time plots of A-PM-TEA/DMF solutions at 1 mg/mL and 20 mg/mL.

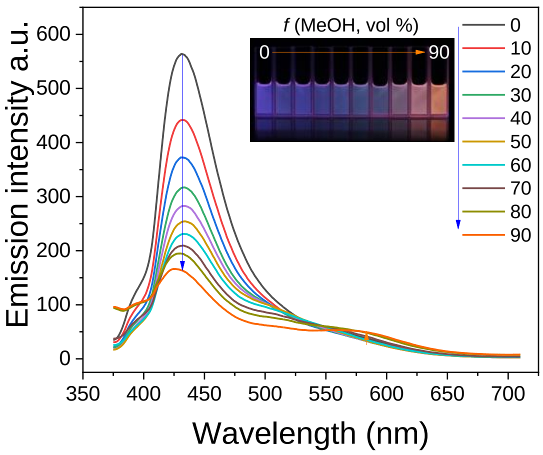

Figure 13 Solvent-dependent emission of A-PM-TEA. Concentration, 10 mg/mL; DMF, good solvent; MeOH, poor solvent.

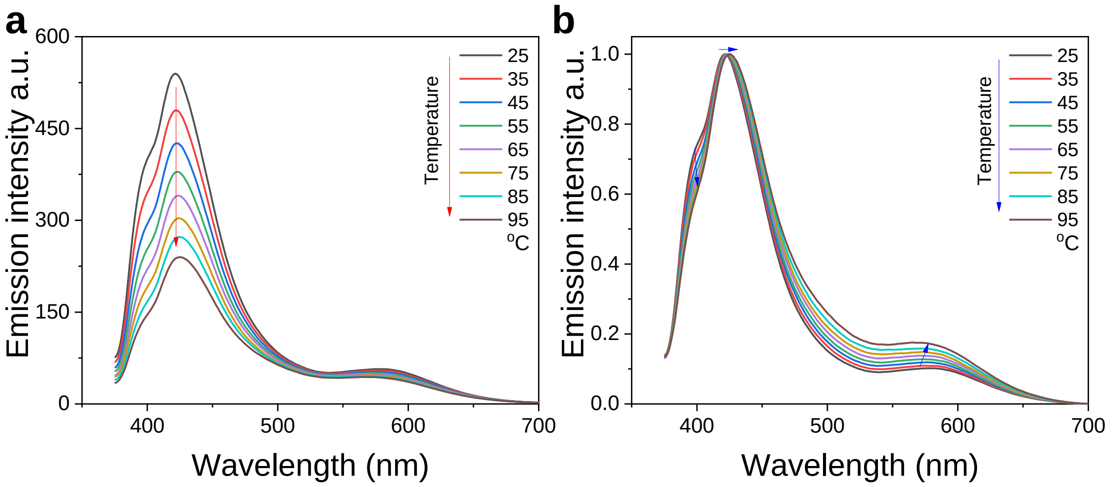

Figure 14 Temperature-dependent emission of A-PM-TEA. a Temperature-dependent emission spectra. **b** Normalized temperature-dependent emission spectra (3 mg/mL, 25-95 °C).

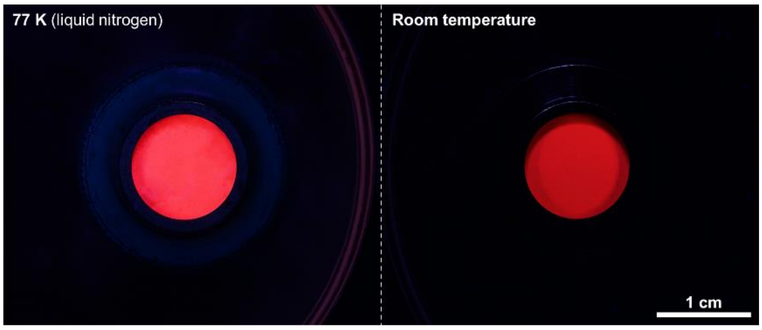

Figure 15 Temperature-dependent fluorescent images of A-PM-TEA powder at 77 K and room temperature respectively.

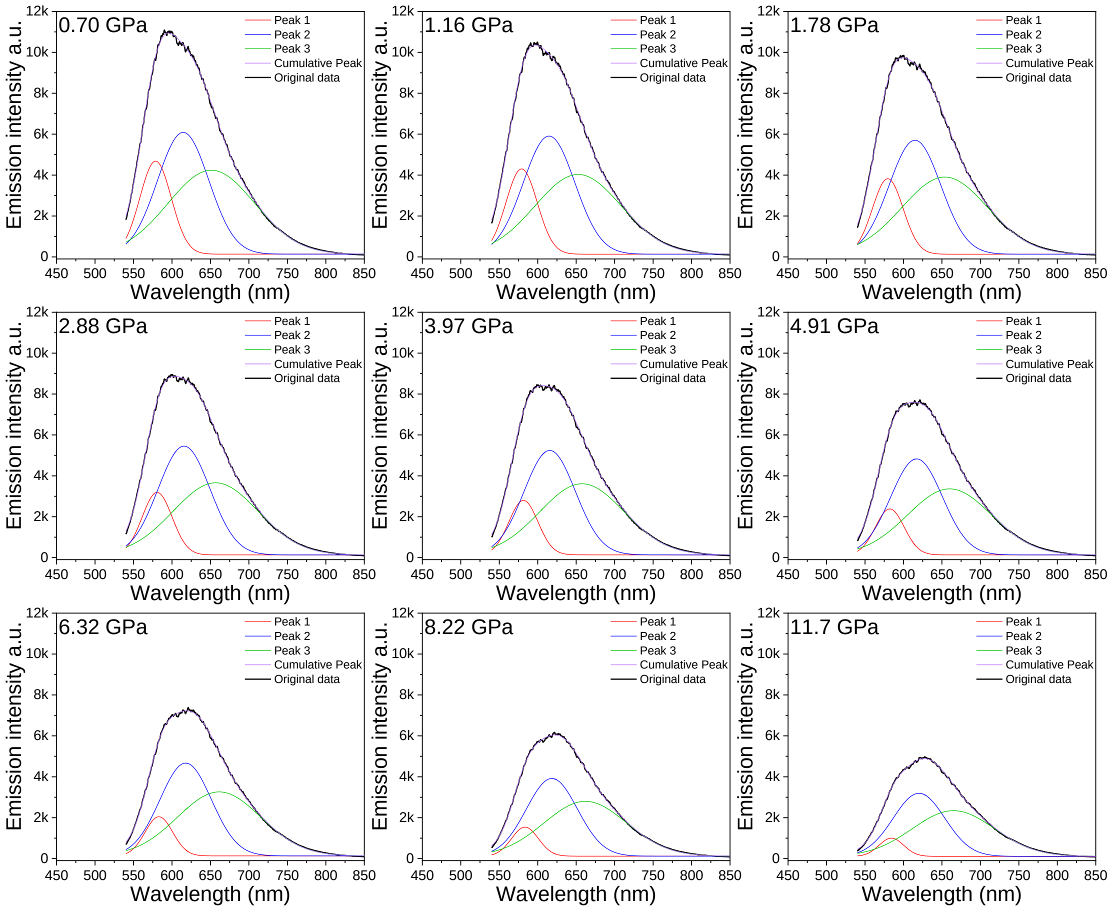

Figure 16 Multi-peak fitting analysis of the emission spectra during compression (0 to 12 GPa)**.**

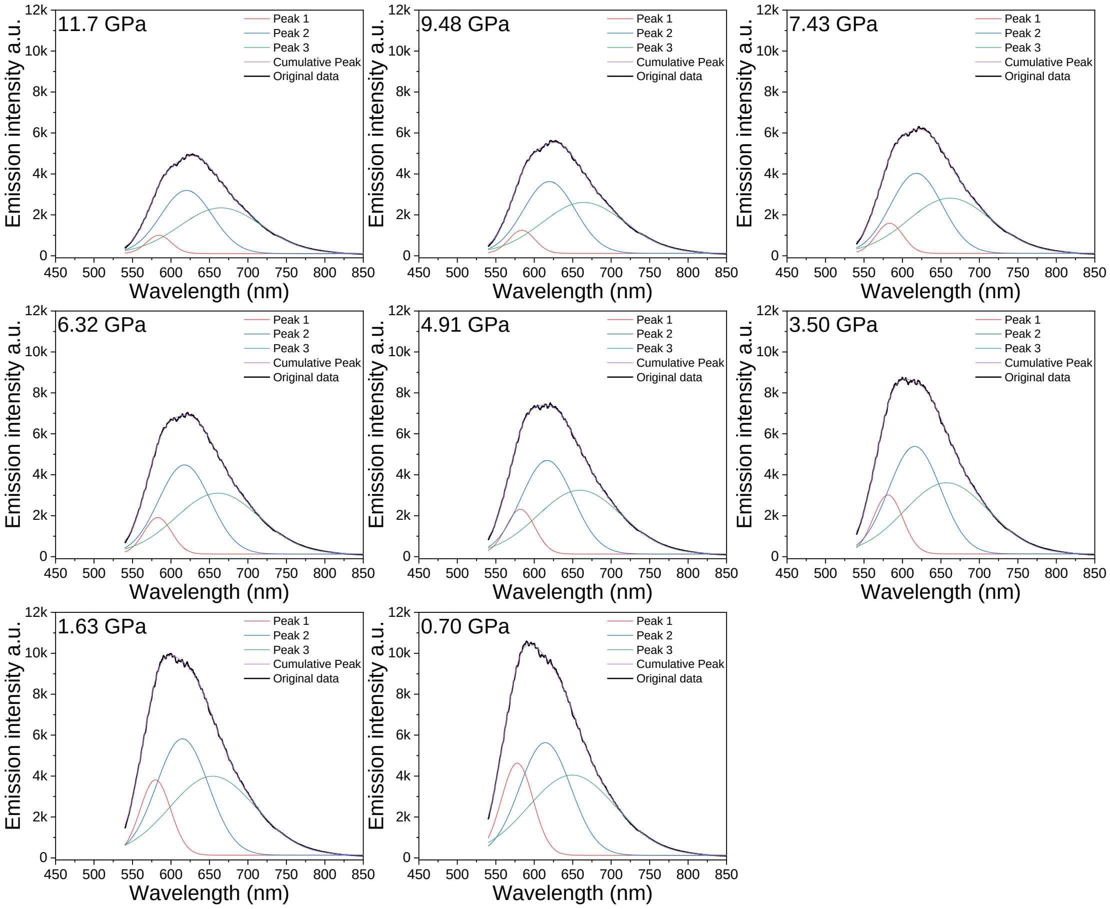

Figure 17 Multi-peak fitting analysis of the emission spectra during decompression (12 to 0 GPa)**.**

.

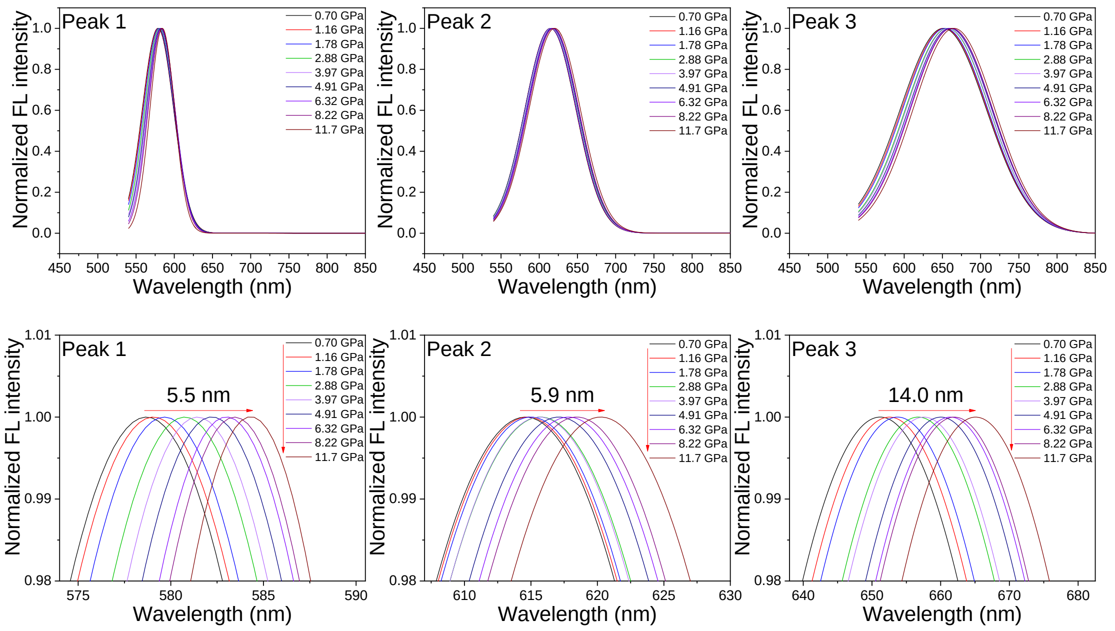

Figure 18 Detailed multi-peak fitting analysis for compression process. Normalized and enlarged views of the underlying peaks (1-3) resolved from the emission spectra of A-PM-TEA under the hydrostatic pressure increasing from 0 to12 GPa.

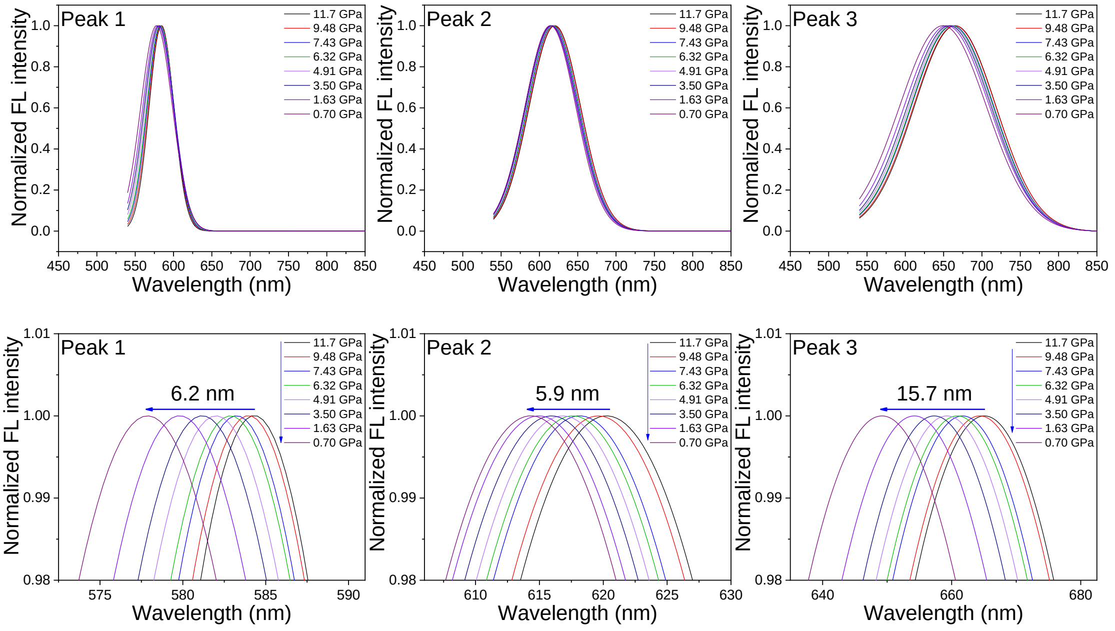

Figure 19 Detailed multi-peak fitting analysis for decompression process. Normalized and enlarged views of the underlying peaks (1-3) resolved from the emission spectra of A-PM-TEA under the hydrostatic pressure decreasing from 12 to 0 GPa.

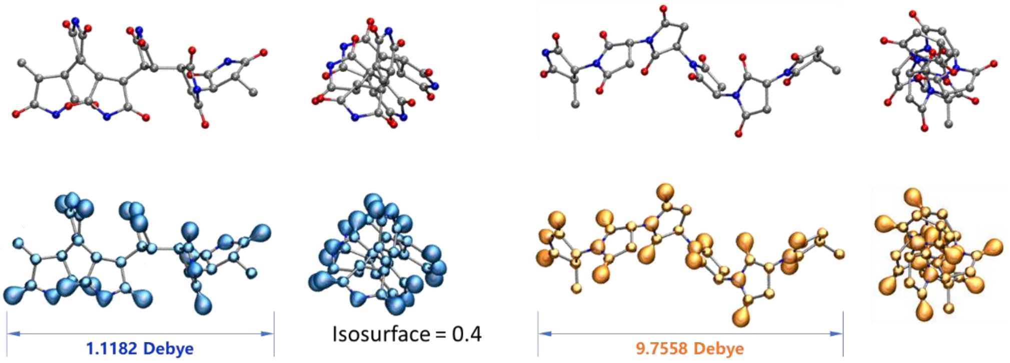

Figure 20 Optimized molecular conformation and dipole moment of PMs (6 repeat units) in -C-C- and -C-N- connections that were calculated using Gaussian 09 with uB3LYP/6-31G(d) package.

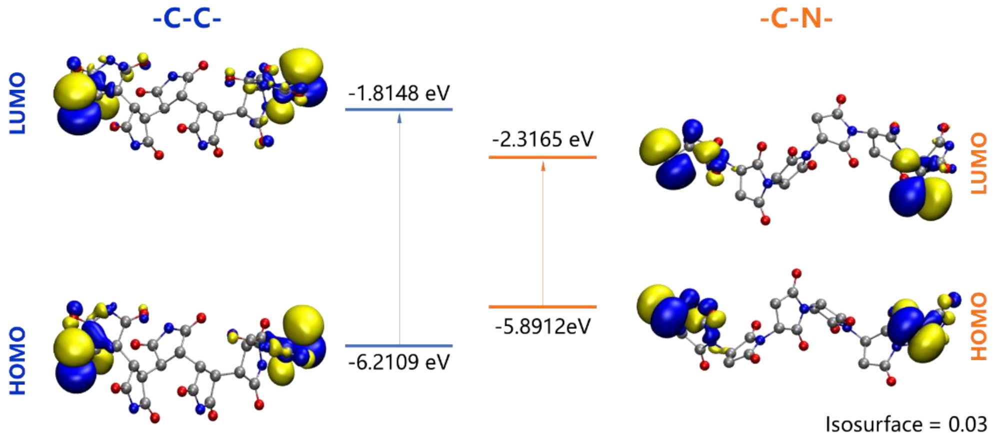

Figure 21 Excited-state HOMO/LUMO of PMs (6 repeat units) in -C-C- and -C-N- connections that were calculated using Gaussian 09 with uB3LYP/6-31G(d) package.

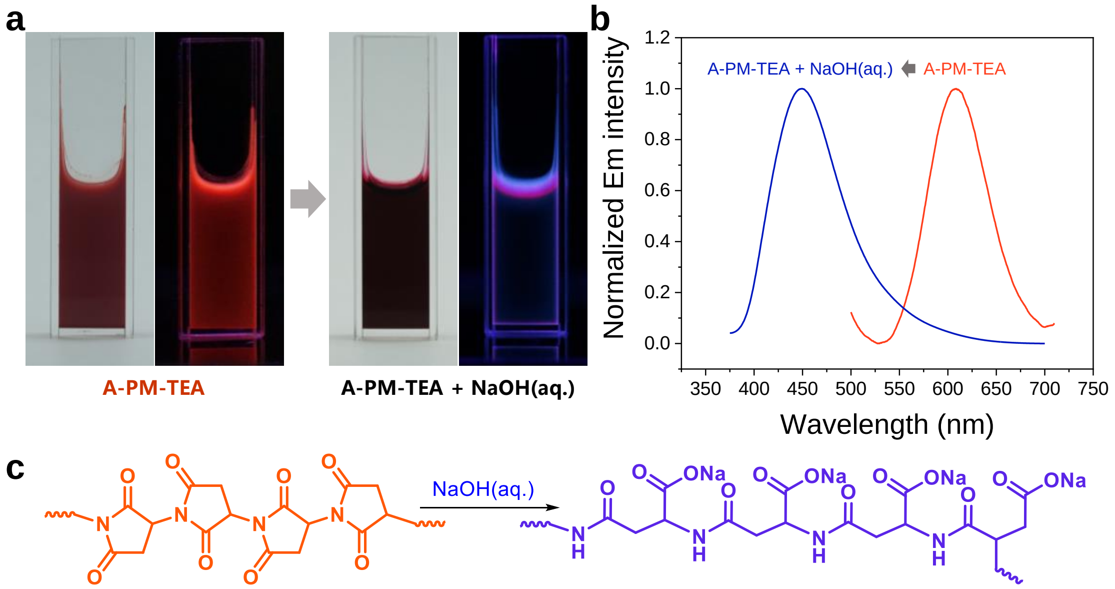

Figure 22 Emission change of A-PM-TEA after hydrolysis. a Images and **b** emission spectra of A-PM-TEA/DMF solution and A-PM-TEA solution after hydrolysis in NaOH (aq.); **c** The schematic hydrolysis of A-PM-TEA in NaOH (aq.).

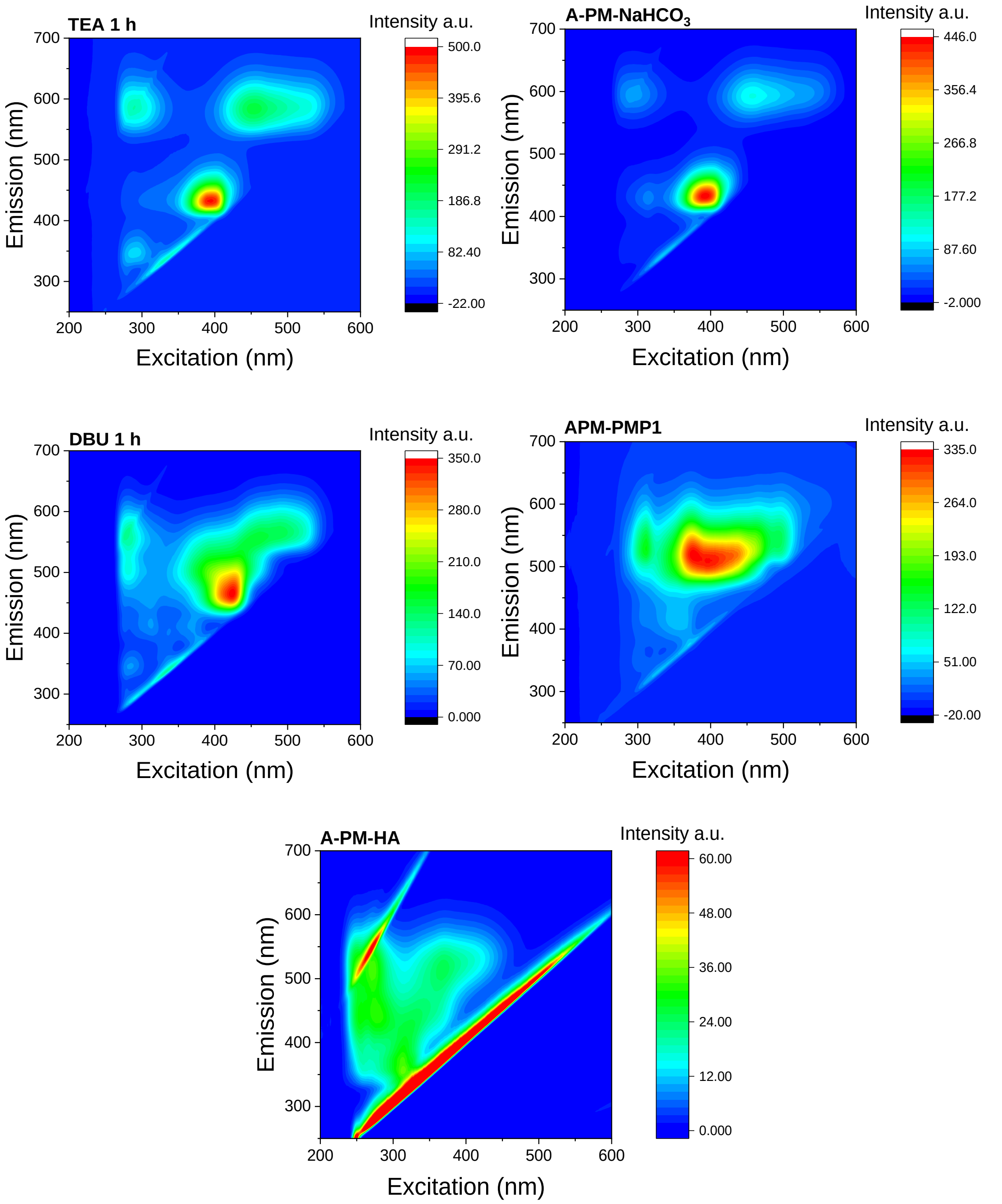

Figure 23 Excitation-emission contour plots of A-PMs. A-PM-TEA/DMF (6 mg/mL), A-PM-NaHCO$_3$/DMF (7 mg/mL), A-PM-DBU/DMF (2 mg/mL), A-PM-PMP/DMF (6 mg/mL) and A-PM-HA/H_2O (10 mg/mL).

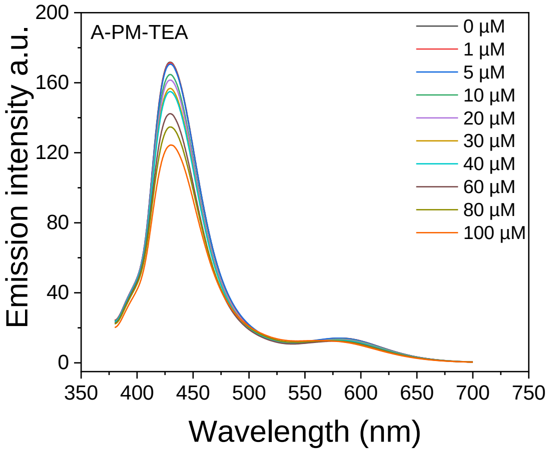

Figure 24 Fluorescent response of A-PM-TEA to Fe^{3+}. Emission spectra of A-PM-TEA/DMF solution (3 mg/mL) with different concentrations of Fe^{3+} (λ_{ex} = 365 nm).

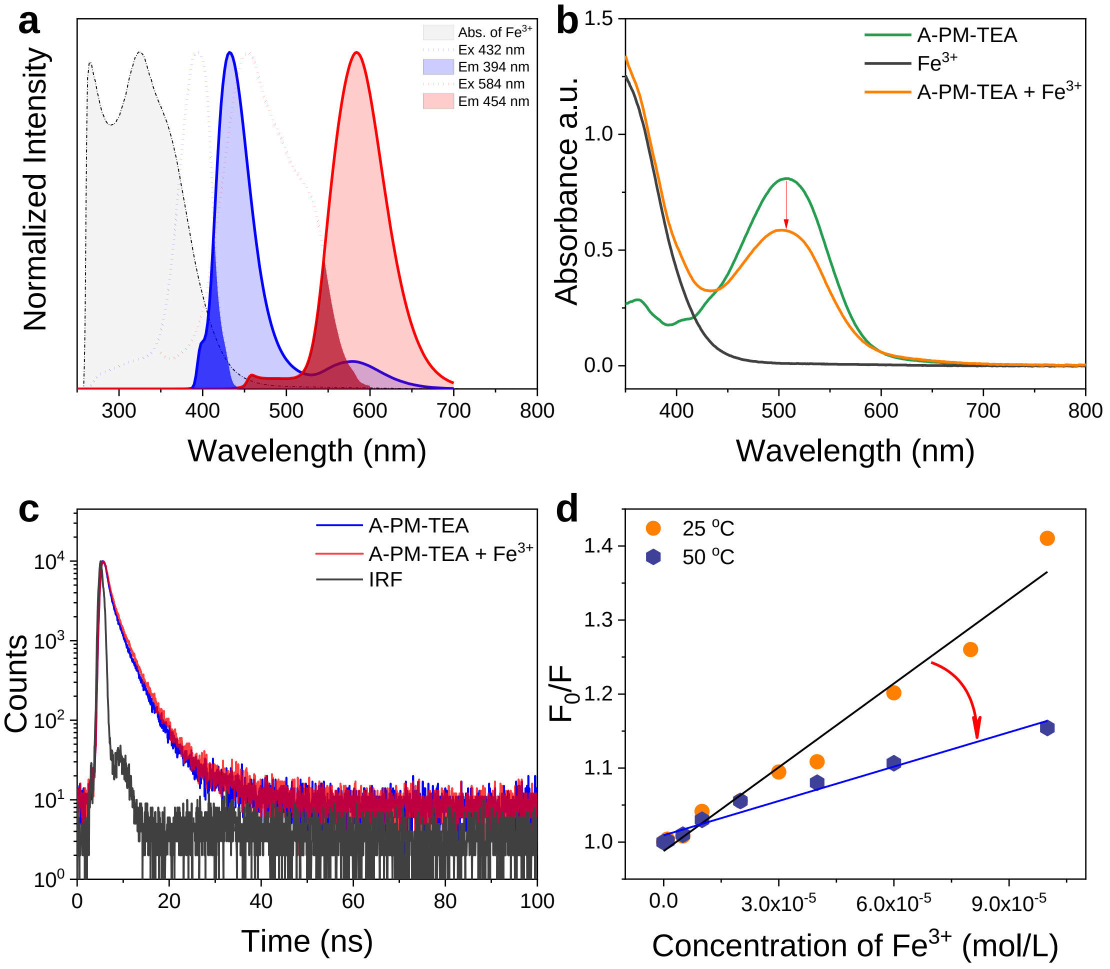

Figure 25 Mechanism for the emission of A-PM-TEA quenched by Fe^{3+}. a Normalized UV-Vis spectrum of Fe^{3+} and normalized excitation/emission spectra of A-PM-TEA solution. **b** UV-vis spectra of Fe^{3+}, A-PM-TEA and A-PM-TEA+Fe^{3+} **c** fluorescence decay curves of A-PM-TEA solution before and after adding Fe^{3+}. **d** Fe^{3+}-concentration dependent plots of F_0/F at different temperatures.

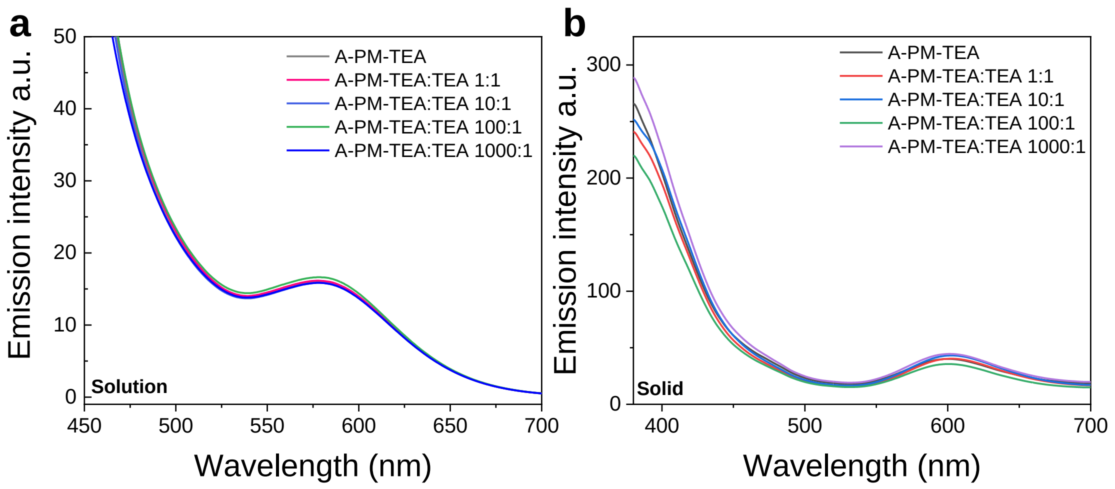

Figure 26 Emission of A-PM-TEA with TEA. a A-PM-TEA/DMF solutions and **b** A-PM-TEA powders mixed with TEA at different concentrations (A-PM-TEA:TEA, mass ratio). Concentration of A-PM-TEA was 5 mg/mL.

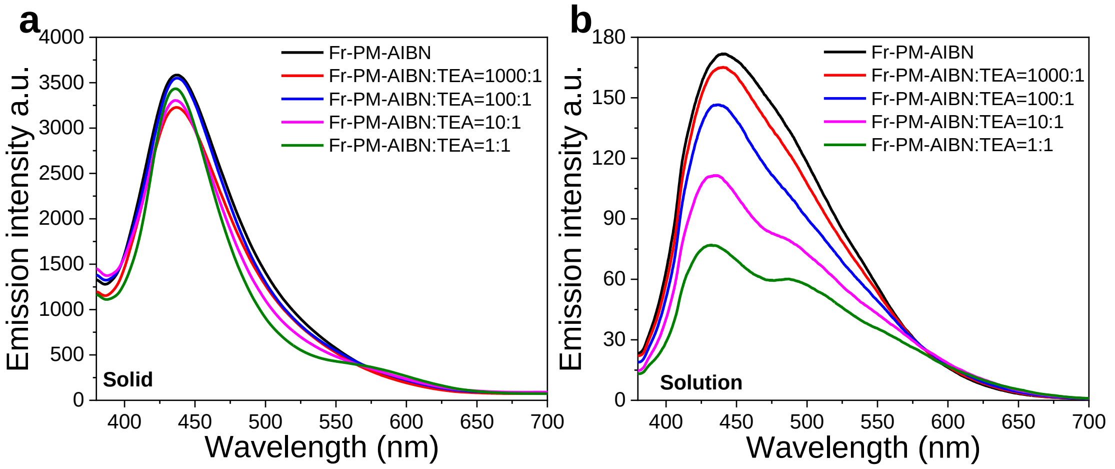

Figure 27 Emission of Fr-PM-AIBN with TEA. a Fr-PM-AIBN powders and **b** Fr-PM-AIBN/DMF solutions mixed with TEA at different concentrations (Fr-PM-AIBN:TEA, mass ratio). Concentration of Fr-PM-AIBN was 10 mg/mL.

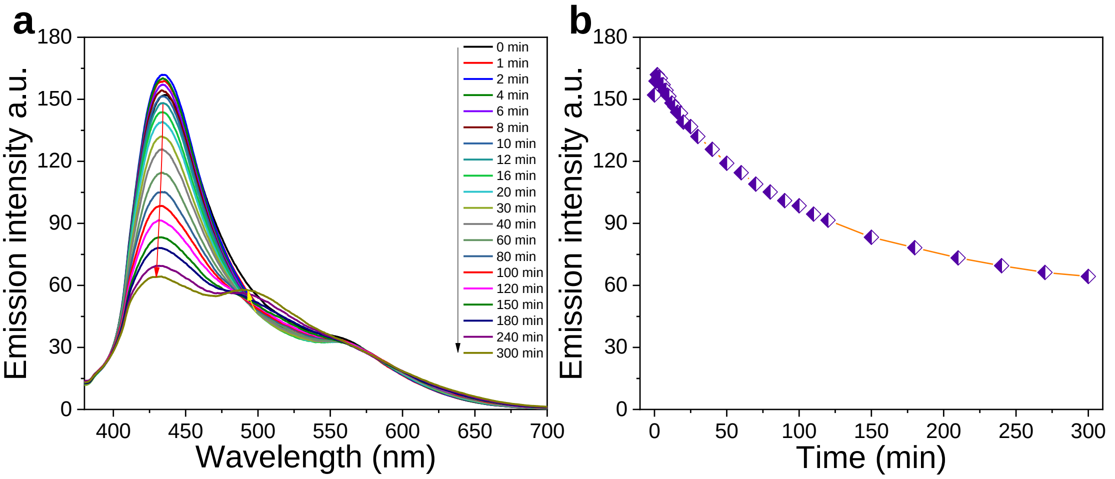

Figure 28 Emission changes of Fr-PM-AIBN/DMF solutions over the time after adding TEA. a Emission spectra**. b** Emission intensity. Concentration of Fr-PM-AIBN was 10

mg/mL and mass ratio Fr-PM-AIBN:TEA = 1:1.

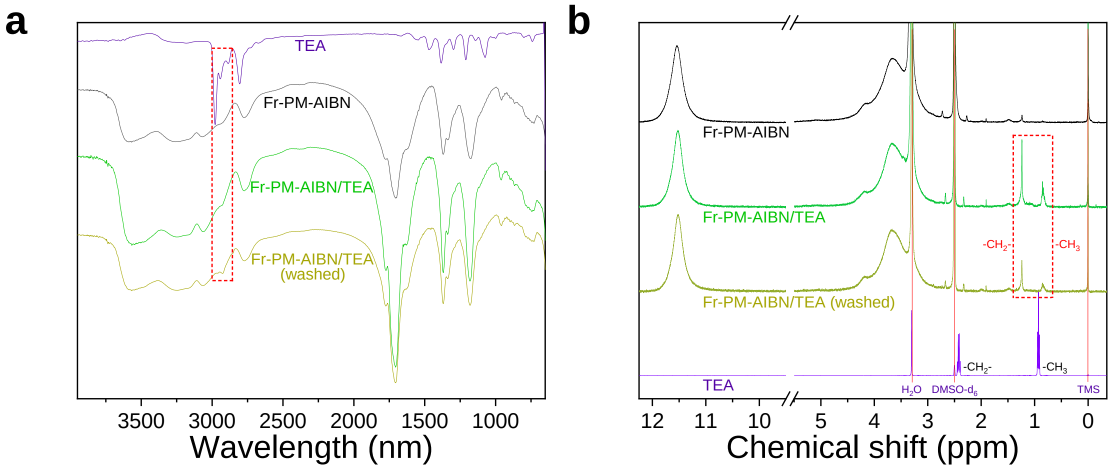

Figure 29 ^{1}H NMR and FTIR analysis of Fr-PM-AIBN before and after mixing with TEA. **a** FTIR spectra. **b** ^{1}H NMR spectra. Mass ratio Fr-PM-AIBN:TEA = 1:1. Fr-PM-AIBN/TEA is the sample from the Fr-PM-AIBN/TEA mixture dissolved in DMSO and recovered in ethanol. Fr-PM-AIBN/TEA (washed) is the sample from the Fr-PM-AIBN/TEA mixture dissolved in DMSO and precipitated in ethanol for 4 cycles.

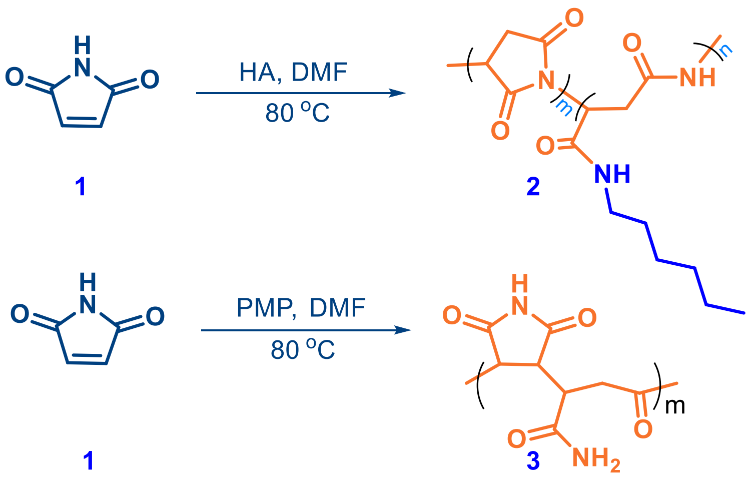

Figure 30 Proposed synthetic routes of A-PM-HA[1] and A-PM-PMP. 1, maleimide; 2, A-PM-HA; 3, A-PM-PMP. Concentration of maleimide was 0.097 g/mL.

Supplementary Tables

Table 1 GPC results of different PMs, including number-average molecular weight (Mn), weight-average molecular weight (Mw), polydispersity (Mw/Mn).

Sample	λ_{max}, nm	Mn	Mw	Mw/Mn
A-PM-HA	464.8	2.053×10^2 (±8.719%)	2.078×10^2 (±8.492%)	1.01
A-PM-PMP	504.0	8.578×10^2 (±1.445%)	1.406×10^3 (±2.681%)	1.64
A-PM-DBU	586.6	9.286×10^3 (±1.024%)	1.062×10^4 (±1.171%)	1.14
A-PM-$NaHCO_3$	596.4	8.844×10^3 (±1.446%)	1.452×10^4 (±1.257%)	1.64
A-PM-TEA	608.6	1.024×10^4 (±0.904%)	1.620×10^4 (±0.801%)	1.58
Fr-PM-AIBN	467.4	3.825×10^4 (±0.567%)	4.927×10^4 (±0.251%)	1.29

Table 2 PLQY % of different A-PM powders (λ_{ex} = 365 nm; initiators, Lewis bases).

No.	Lewis base	PLQY %
I	TEA	1.2
II	$NaHCO_3$	1.4
III	DBU	1.5
IV	PMP	3.6
V	HA	16.2

Table 3 GPC analysis of the polymerization kinetics for A-PM-TEA. Number-average molecular weight (Mn), weight-average molecular weight (Mw), polydispersity (Mw/Mn) of PMs synthesized with TEA for different reaction time (80 °C, 10-360 min).

Reaction time (min)	Mn	Mw	Mw/Mn	PLQY %
10	2.501×10^3	3.528×10^3	1.411	1.1 %
20	3.333×10^3	4.188×10^3	1.256	1.3 %
30	3.884×10^3	4.941×10^3	1.272	1.4 %
40	4.414×10^3	5.430×10^3	1.230	1.4 %
50	5.312×10^3	6.231×10^3	1.173	1.3 %
60	5.594×10^3	6.603×10^3	1.180	1.2 %
120	6.716×10^3	8.483×10^3	1.263	1.1 %
180	7.832×10^3	1.047×10^4	1.336	0.9 %
360	1.361×10^4	2.233×10^4	1.641	0.7 %

Table 4 PLQY of A-PM-TEA/TEA mixtures (powders) at different TEA concentrations.

TEA concentration, wt.%	PLQY %
50.0	0.5 %
10.0	0.4 %
1.0	0.5 %
0.1	0.4 %
0	0.4 %

Table 5 Synthetic information of the samples presented in the main text.

Figures	Sample	initiator	Temperature (°C)	Time (h)	[monomer]/[initiator] (molar)	yield %
Fig. 1a	Fr-PM-AIBN	AIBN	60	24	1:1.95	72
	A-PM-TEA (s)	TEA	80	1	10:1	61
	A-PM-PMP (s)	PMP	80	1	10:1	49
	A-PM-HA (s)	HA	80	1	1:1	55
	A-PM-TEA (l)	TEA	80	12	10:1	/
	A-PM-PMP (l)	PMP	80	6	10:1	/
	A-PM-HA (l)	HA	80	2	1:1	/
Fig. 2a-d	A-PM-TEA (l)	TEA	80	12	10:1	/
	A-PM-$NaHCO_3$ (l)	$NaHCO_3$	80	1	10:1	/
	A-PM-DBU (l)	DBU	80	1	10:1	/
	A-PM-PMP (l)	PMP	80	6	10:1	/
	A-PM-HA (l)	HA	80	2	1:1	/
	A-PM-TEA (s)	TEA	80	1	10:1	61
	A-PM-$NaHCO_3$ (s)	$NaHCO_3$	80	1	10:1	67
	A-PM-DBU (s)	DBU	80	1	10:1	70
	A-PM-PMP (s)	PMP	80	1	10:1	49
	A-PM-HA (s)	HA	80	6	1:1	55
Fig. 3a-b	A-PM-TEA	TEA	80	6	10:1	65
Others	Fr-PM-AIBN	AIBN	60	24	1:1.95	72
	A-PM-TEA	TEA	80	1	10:1	61
	A-PM-$NaHCO_3$	$NaHCO_3$	80	1	10:1	67
	A-PM-DBU	DBU	80	1	10:1	70
	A-PM-PMP	PMP	80	1	10:1	49
	A-PM-HA	HA	80	1	1:1	55

Note: (s), solid powder; (l) solution.

Supplementary Methods

Cell viability assay for skin-contact compatibility. 1.0 g of A-PM-TEA powder was soaked in 5 mL of Dulbecco's Modified Eagle Medium (DMEM; Gibco, USA) for 24 h. Then the mixture was filtered with a syringe-driven filter (0.2 μm) and the extracted solution was diluted with DMEM in a concentration gradient (100%, 75%, 50%, 25%, 10%, 1%). The human epidermal keratinocyte line HaCaT cells were cultured in DMEM containing 10% Fetal Bovine Serum (FBS; Gibco, USA), 100 U/mL penicillin and 100 μg/mL streptomycin (Penicillin-Streptomycin-Glutamine; Gibco, USA) with a Forma Steri-Cycle CO_2 incubator (Thermo Fisher Scientific, USA, 37 °C, 5% CO_2, humid atmosphere) for 24 h. Finally, the HaCaT cells were respectively immersed in the previous extracted solutions (100%, 75%, 50%, 25%, 10%, 1%) for 15 min and the Cell Counting Kit-8 (CCK-8, NCM, China) test was conducted to assay cell viability of HaCaT.

Fluorescent pattern printing. The trichromatic fluorescent printing inks were prepared by simply blending the powders of PMs, including A-PM-TEA, A-PM-PMP and A-PM-HA into a small amount of DMF. Cellulose acetate and glycerol were added as thickening and wetting agents for printing inks. Mandala patterns were printed via manual multi-color screen printing technology as follows. First of all, the screens with patterns for different ink colors (red, green, blue) were cured with light-sensitive emulsion. Secondly, one screen for a single ink color and printing substrate (paper) were mounted securely and accurately. Then the printing ink was poured on one side of the screen and a rubber squeegee was used to push the ink from one side to another so that a color pattern was printed on the paper. Finally, repeating above process with different screens and color inks, a multi-color Mandala pattern was obtained.

Metal ions detection. Aqueous solutions (1 mM/L) of metal ions (Ag^+, Ba^{2+}, Ca^{2+}, Cd^{2+}, Co^{2+}, Cr^{3+}, Cu^{2+}, Fe^{2+}, Fe^{3+}, K^+, Mg^{2+}, Mn^{2+}, Na^+, and Ni^{2+}) and A-PM-TEA/DMF solution (3 mg/mL) were prepared in advance. 0.05 mL of each metal ion solution was added into 0.95 mL of A-PM-TEA/DMF solution separately and emission spectra of the mixed solutions were recorded to determine the selectivity for metal ions detection.

Furthermore, a series of Fe^{3+} aqueous solutions with different concentration (20-2000 μM) were prepared. 0.05 mL of each Fe^{3+} solution was dropped into 0.95 mL of A-PM-TEA6/DMF solution separately and emission intensity variation of the mixed solutions were recorded to determine the LOD (limit of detection) of limit of Fe^{3+}. The LOD (9.54 uM) is given by the linear plots with the following equation [2]:

$$\mathrm{LOD} = \frac{3 \times s_{\mathrm{B}}}{a} \qquad (1)$$

where s_B is the standard derivation of the blank sample and a is the slope of emission intensity variation plot.

Latent fingerprint detection. The latent fingerprint acquisition was conducted according to a standard procedure of powder technology and the ultrafine powder of A-PM-TEA prepared by spray-drying was utilized. The fingerprints of volunteer were collected as following steps. The volunteers thoroughly washed their fingers first and then gently rubbed their fingers over forehead or nose for several times, finally pressed their fingers on different substrates to develop fingerprints. Subsequently, a soft brush that had been dipped in the previous ultrafine powder gently swept over the substrate and sprinkled the powder on the fingerprint. The excess loose powder was removed with a rubber suction bulb. In the end, the fluorescent images of the well-developed fingerprints were recorded with a digital camera under 365 nm ultraviolet light.

Supplementary Discussion

Through space charge transfer (TSCT), intermolecular charge transfer (ICT) and internal conversion (IC). A closer intermolecular distance under high pressure is beneficial to through-space charge transfer (TSCT) and enhanced TSCT and ICT can significantly reduce the HOMO/LUMO energy gap (*ΔE*). According to the equation between the rate of internal conversion (k_{ic}) and the energy gap *ΔE*,

$$k_{\mathrm{ic}} = 10^{13}\mathrm{e}^{-\mathrm{a}\Delta E} \quad (1)$$

A smaller ΔE gives a higher k_{ic} and can intensify the non-radiative decay of the excited singlet state because of the coupling of rotational and vibrational energy-levels. The photoluminescence quantum yield (Φ) can be obtained by following functions.

$$\Phi = k_{\mathrm{f}}/(k_{\mathrm{f}} + k_{\mathrm{ic}}) \propto 1/k_{\mathrm{ic}} \quad (2)$$

$$\Delta E = \mathrm{h}\nu = \mathrm{h}/\lambda \quad (3)$$

$$\Phi \propto 10^{13}\mathrm{e}^{a\mathrm{h}/\lambda} \quad (4)$$

There is an exponential relationship between PLQY (Φ) and λ_{max} of emission, which exactly coincides with the exponential decline plot of PLQY in Supplementary Figure 7.

Generally, the emission spectra of the charge transfer complexes from TSCT and ICT are strongly influenced by the polarity of solvent [3]. The red shift of emission spectra is always observed with increasing the polarity of the solvent. Such a red shift of λ_{max} is also observed in Figure 3c-d in manuscript.

Mechanism for the emission of A-PM-TEA quenched by Fe^{3+}. As shown in Figure S25a-b, there are obvious overlaps between the absorption spectra of Fe^{3+}, A-PM-TEA+Fe^{3+} and the excitation/emission spectra of A-PM-TEA. Therefore, the inner filter effect is inevitable for emission quenching of A-PM-TEA by adding Fe^{3+}.

In addition, the fluorescence lifetime of A-PM-TEA (Supplementary Figure 25c) does not change after adding Fe^{3+}. The slope of the Fe^{3+}-concentration dependent F_0/F plots becomes smaller when rising the temperature from 25 to 50 °C (Supplementary Figure 25d). These results indicate that the static quenching mechanism is involved in A-PM-TEA+ Fe^{3+} system.

Conclusively, the emission quenching of A-PM-TEA by adding Fe^{3+} should be a synergic result of the inner filter effect and the static quenching mechanism due to the formation of A-PM-TEA-Fe^{3+} complexes.

Emission of A-PM-TEA and Fr-PM-AIBN with the residual TEA

The potential complex between maleimide units and the trace amount of Lewis bases (amine groups) should be concerned to demonstrate red emission of the nonconventional luminescent PMs. However, the interference of A-PM-TEA/TEA complex can be excluded from our systems on the basis of following experimental evidences.

1. The molecular structure and luminescent characteristics of A-PM-TEA and A-PM-$NaHCO_3$ (major repeat units, -C-N-; orange red emission) are very close. It is impossible that red emission of A-PM-$NaHCO_3$ originates from the maleimide-amine complex, because TEA is not involved in A-PM-$NaHCO_3$. In addition, different from the organic Lewis base TEA, the inorganic $NaHCO_3$ can be easily removed by water washing. Therefore, the interference of $NaHCO_3$ is also excluded.

2. If the red emission is caused by the complex between maleimide units and the trace amount of amines, the addition of TEA into A-PM-TEA solution or powders would enhance n red emission because of the increased maleimide-amine complexes. However, as seen in Supplementary Figure 26, different amount of TEA was blended into A-PM-TEA/DMF solutions or A-PM-TEA powders and no obvious changes were observed in their emission spectra. The PLQY of A-PM-TEA/TEA mixture (powders) in Supplementary Table 4 indicates that the addition of TEA has little influence on the emission of A-PM-TEA, considering the instrumental error for powder tests.

Above experimental results confirm that the red emission of A-PM-TEA should be attributed to its polymer structure rather than the residual maleimide-amine complexes.

Same experiment in Supplementary Figure 26 is conducted for Fr-PM-AIBN/TEA mixtures. As shown in Supplementary Figure 27a, the emission spectra of Fr-PM-AIBN powder have changed little after mixing with TEA in different mass ratios, indicating a limited influence of TEA on the emission of Fr-PM-AIBN powders. However, the emission of Fr-PM-AIBN/DMSO solutions in Supplementary Figure 27b seem sensitive to TEA and are gradually quenched by increasing the concentration of TEA in solutions. As suggested, the emission spectra of Fr-PM-AIBN/TEA solutions over the mixing time were recorded. The results in Supplementary 28a-b show a continuous decline in emission intensity after a sudden rise at the beginning. Interestingly, a new long-wavelength peak at approximately 500 nm indeed appears, which is obviously different from the result in Supplementary Figure 26. This new emission peak and the emission quenched by TEA could be ascribed to the formation of new luminescent species, the Fr-PM-AIBN/TEA complex, which can be further confirmed by FTIR and ^1H NMR analysis. FTIR in Supplementary Figure 29a shows that the characteristics of $-CH_2-$, $-CH_3$ (2930, 2970 cm^{-1}) appear in the FTIR of Fr-PM-AIBN/TEA samples in comparison with Fr-PM-AIBN. More evidently, peaks of $-CH_2-$ and $-CH_3$ (1.24, 0.84 ppm) that are different from that of the small molecular TEA (2.41, 0.92 ppm) emerges from the ^1H NMR spectra of Fr-PM-AIBN/TEA samples. These characteristic peaks are absent in the ^1H NMR of Fr-PM-AIBN and cannot be eliminated by washing the sample repeatedly. Comparatively, the minimal changes in emission of Fr-PM-AIBN powders with TEA are attribute to the slow kinetic rate of solid-phase interactions.

The emission of A-PM-AIBN/TEA suggests that the blue intrinsic emission of Fr-PM-AIBN also originates from its -C-C- polymer structure, instead of the complex of Fr-PM-AIBN and residual chemicals, which generally has a longer wavelength of emission.

Supplementary References

1. Xu, W. et al. Amphiphilic hexylamine modified polysuccinimide: Synthesis, characterization, and formation of nanoparticles in aqueous medium. *J. Macromol. Sci., Pure Appl. Chem.* **A40**, 511-523 (2003).
2. Miller J, M. J. C., *Statistics and chemometrics for analytical chemistry*. (Pearson education, London, 2018).
3. Lakowicz, J. R., *Principles of Fluorescence Spectroscopy*. (Springer Science+Business Media, New York, 2006).
